# P01-029 – Microscopic hematuria in FMF

**DOI:** 10.1186/1546-0096-11-S1-A33

**Published:** 2013-11-08

**Authors:** L Grabowitz, OL Kukuy, M Lidar, I Ben Zvi, O Feld, Y Kesel, A Livneh

**Affiliations:** 1 Sackler School of Medicine, Tel Aviv University, Tel Aviv, Israel; 2 Heller Institute of Medical Research, Israel; 3 Institute of Nephrology and Hypertension, Sheba Medical Center Tel HaShomer, Ramat Gan, Israel; 4 Medicine F, Sheba Medical Center Tel HaShomer, Ramat Gan, Israel

## Introduction

Hematuria is a recognized feature of familial Mediterranean fever (FMF), but its prevalence and clinical, genetic and demographic correlates are not known.

## Objectives

To study the rate and features of microscopic hematuria in FMF.

## Methods

We studied consecutive FMF patients, who came for a pre-scheduled follow up visit in the FMF clinic for the presence of microscopic hematuria, defined as ≥5 RBC/HPF or ≥25RBC/µl in urine analysis performed during remission, recorded at least once in the 3 previous clinic visits. Exclusions were known kidney, urinary tract, prostate or gynecologic diseases, bleeding or thrombotic diatheses, pregnancy or menstruation, intensive physical activity and anticoagulant/platelet treatments. Patients presenting with hematuria were compared to patients without hematuria for various clinical, genetic and demographic parameters, using a questionnaire, patient files, and an interview.

## Results

The frequency of microscopic hematuria among FMF patients was found to be 17% (30/173), not conspicuously higher than in the general population (1-16%). Hematuria was associated with higher levels of acute phase reactants during the attack-free phase, and higher rates of a history of vasculitides: protracted febrile myalgia and Henoch Schonlein Purpura. There were no differences in the distribution of severity scores among patients of the hematuria and control groups. The rate of homozygosity to M694V and the rate of 2 affected MEFV alleles was similar to that of the control group.

## Conclusion

This study could not confirm the notion that microscopic hematuria is more common in FMF. However, its occurrence may reflect an active disease and renal vascular inflammation

## Disclosure of interest

None declared.

